# A Rare Case of Recurrent Hematuria from Right Kidney: Radiologic Diagnosis and Treatment

**DOI:** 10.5402/2011/159104

**Published:** 2011-07-19

**Authors:** Pietro Venetucci, Mario Quarantelli, Vittorio Iaccarino

**Affiliations:** Dipartimento Assistenziale di Diagnostica per Immagini, Sezione di Radiologia Cardiovascolare ed Interventistica, Università degli Studi di Napoli, A.O.U. Policlinico “Federico II”, Via Pansini, 5-80131, Napoli, Italy

## Abstract

We report the case of a young woman admitted because of several and recurring episodes of macroscopic hematuria beginned after her first pregnancy. Contrast-enhanced multidetector computed tomography images showed dilated ovarian veins due to a typical pelvic varicocele. We supposed to be a right ovarian vein syndrome, a rare clinical situation characterized by an anomalous compression of the lumbar ureter by the ectasic ovarian vein; this condition may cause a chronic inflammatory stimulus above the urothelial mucosa with a following hematuria. All symptoms were solved by an endovascular treatment through the sclero-embolisation of the pelvic varicocele. After eighteen months the patient didn't present hematuria anymore and she no longer complained about her right side lumbar pain.

## 1. Introduction

Pelvic varicocele is the cause of the so-called “pelvic congestion syndrome”, a quite frequent clinical condition in which the ectatic veins cause a congestion of all the pelvic organs; this pathology is very often underestimated and misdiagnosed because it is characterized by some aspecific and vague symptoms, but sometimes invalidant, that make the diagnosis very difficult. On the contrary, the development of a right ovarian vein syndrome is a very rare clinical entity; this pathology is thought to sometimes occur when the pelvic varicocele is associated to a dilation of the right ovarian vein; the lumbar ureter at the crossing with the vascular iliac trunk is trapped into a kind of “vascular forceps” generated by the common iliac artery and the ectatic ovarian vein. The same ovarian veins dilation occurs, for example, after the assumption of a high dose of estrogens or for other reasons. The compression of the ureteral lumen may cause some indefinite symptoms up to serious clinical manifestations; the decubitus of the ureteral walls generates a chronic inflammation of the urothelial mucosa with a plicar hypertrophia that produces recidivant lumbar and pelvic pain, as a renal colic. In this condition, we can find an aseptic leucocyturia; the pyelonephritis developing and rare episodes of urinary bleeding could finally occur.

We have not found in the literature any analogous cases in which the hematuria has been the first clinical manifestation of this rare syndrome in absence of typical pelvic pain and, above all, any experience about the radiologic endovascular management of the symptoms.

## 2. Case Report

We received a request of consultation for a 32-year-old woman with a 5-year history of frequent and recurring episodes of gross hematuria.

Urinary bleeding was present without dysuria and was typically spontaneous and intermittent. The patient was admitted four years before in another university hospital of the North of Italy where she underwent several diagnostic procedures; among these, a cystoscopic evaluation showed a right ureteral hematuria and the ureteroscopy, with the associated mucosal brushing of the lumbar tract, indicated an aspecific phlogistic reaction with the presence of only inflammation cells.

**Figure 1:**
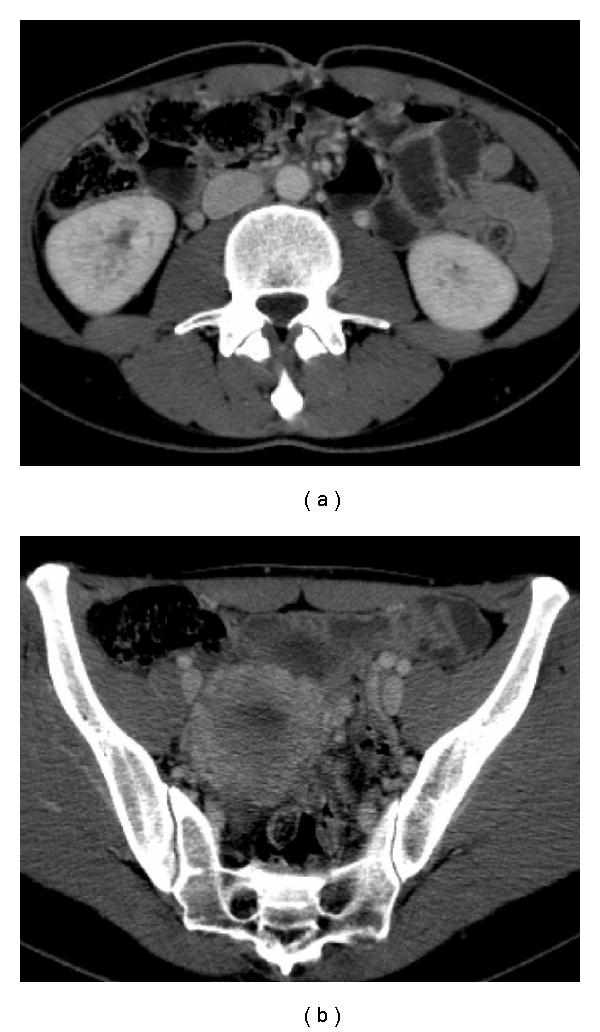
Retrospective evaluation of CT images showed a great dilation of the ovarian veins and of the periuterine venous plexus.

**Figure 2:**
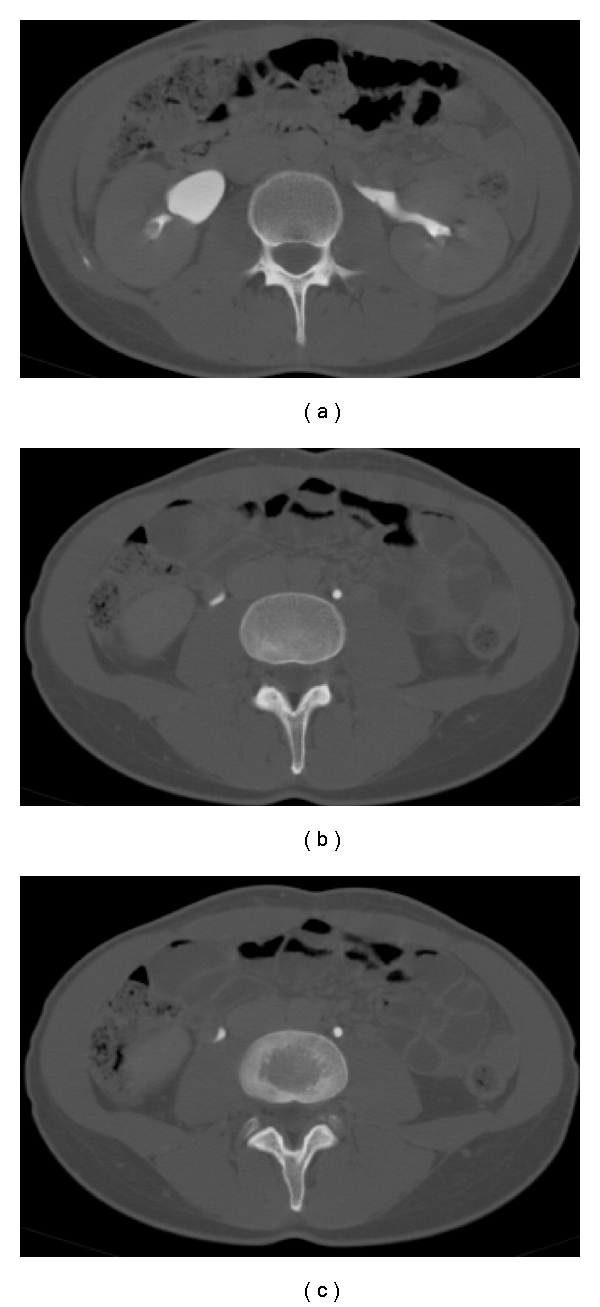
The right ovarian vein is in a very close position to the lumbar ureter, at the crossing with the iliac artery, where it appears to have an irregular morphology and a sharpened caliber; we also note a pielic dilation.

**Figure 3:**
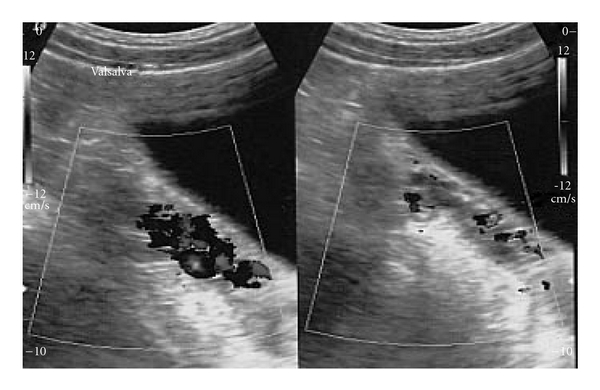
Great dilation of the periuterine venous plexus with blood reflux.

In addition, a subsequent renal biopsy was completely negative. The research of the Koch's bacillus on the urinary culture was also negative. Without a clear diagnosis and, above all, with a persistent hematuria, the patient was admitted in 2007 to our hospital where she practiced a sequential renal scintigraphy and a multidetector contrast-enhanced computed tomography, both negative. The patient was sent to our consultation. In the retrospective CT images, we discovered only dilated ovarian veins (Figure 1). In fact, the delayed contrast phase performed during the renal excretion with the help of 3D reformatted images, indicated an irregular right ureteral morphology with a sharpening of its lumen due to an external compression where it crossed the right iliac artery and the correspondent ovarian vein. A slight pielic enlargement with a dilation of the proximal right ureter was also associated. Finally, we appreciated some peri uterine and periovarian varices, like a pelvic varicocele (Figure 2) and a pelvic ultrasonographic examination with a color-Doppler evaluation which confirmed our diagnosis (Figure 3). So we supposed that a right ovarian vein syndrome could have caused the hematuria. Our diagnosis was confirmed by a subjective symptom during our anamnesis with pain referred only on the right iliac fossa and independent from the decubitus. We did not find any clinical symptom of pelvic congestion (no dull pelvic pain), neither lower extremity venous insufficiency.

## 3. Materials and Methods

**Figure 4:**
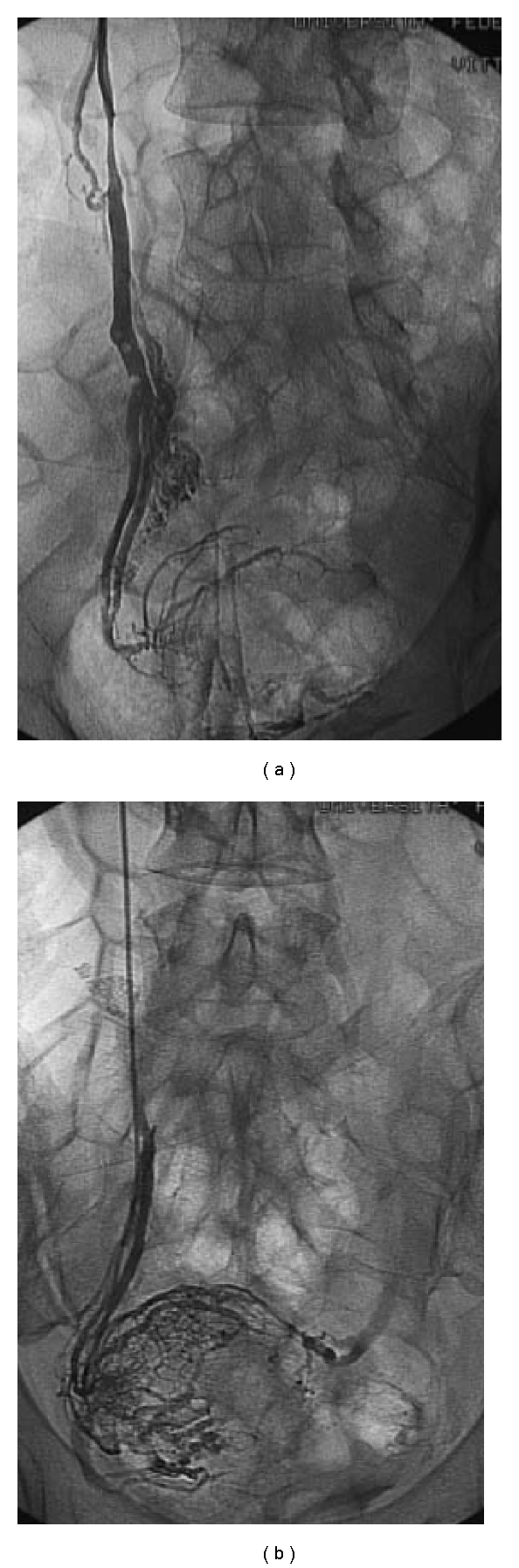
On the left side, we practiced a complete venous sclerosis, while on the right side the treatment has been limited to the distal tract.

**Figure 5:**
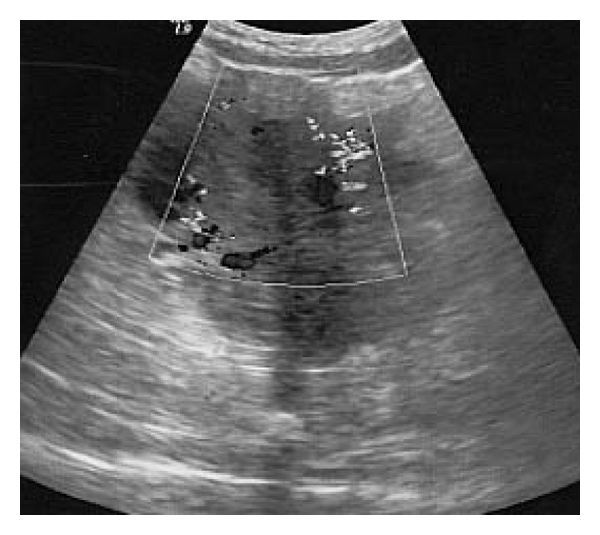
Color-Doppler evaluation shows the decreasing of the periuterine Varices.

**Figure 6:**
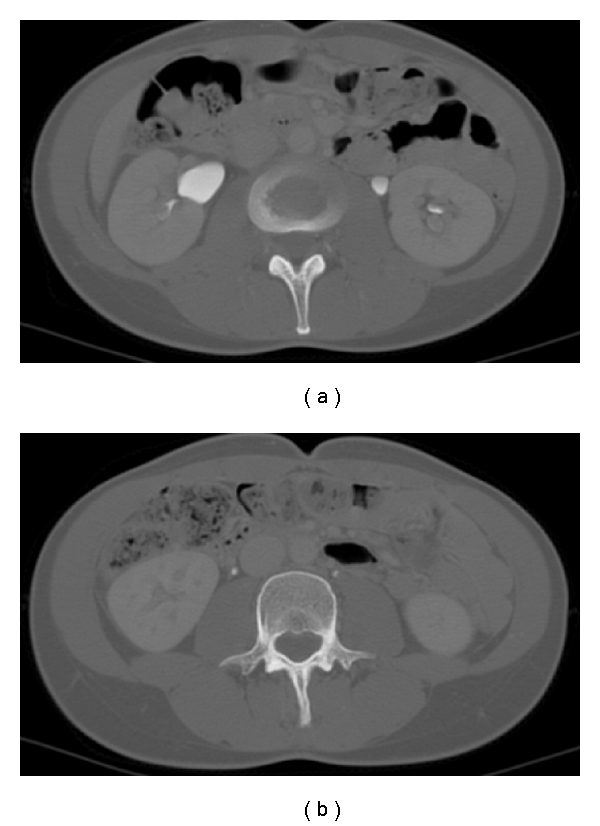
CT evaluation shows the decreasing of the pielic dilation with a good caliber and a normal ureteral morphology.

The patient accepted to undergo an endovascular treatment of the pelvic varicocele. After informed consent, by a percutaneous common femoral vein access, we performed a catheterization and a subsequent selective phlebography of both the ovarian veins. Like in the male varicocele, we used the scleroembolisation technique, injecting a sclerosing agent (sodium tetradecilsolphate 3%). A complete sclerosis of the ovarian vein was first performed on the left side while, on the right one, only a sclerosis of the distal third, saving the lumbar tract where endoscopic evaluation had just showed a phlogistic reaction (Figure 4). The treatment was successfully performed and the color-Doppler evaluation was unable to depict the periuterine varices anymore (Figure 5). CT examination confirmed the decreasing of the pielic and ureteral dilation with a good return to a normal caliber and morphology (Figure 6). During the following 3 months, the patient was completely free from right-side pain and, at the followup at 1st, 6th, 12th, 18th, and 28th month, she never suffered hematuria anymore.

## 4. Discussion

The pelvic varicocele is very often unknown and underestimated but it may cause a lot of psychological and social problems to the young affected patients [1].

The symptoms are quite aspecific and vague: chronic pelvic heaviness, especially referred after a long standing position, for example, after a long working day, dyspareunia, dysmenorrhea, menorrhagia, urinary urgency, and constipation [2]. In a great number of cases, a lot of diagnostic examinations are performed, from endoscopic (cystoscopy, ureteroscopy) to radiologic ones (scintigraphy, CT scan) and the discovering of a pelvic varicocele is a casual event. Instead, a simple pelvic u.s. Doppler could be the only fast and sufficient diagnostic procedure. Frequently the radiologic signs of this pathology, the periuterine varices, are not considered a pathological finding and are not depicted by the radiologist either [3, 4]. In our case, the hematuria was the only objective symptom referred by the patient and it is thought to be very unusual. On the contrary, the only subjective symptom was the right iliac fossa pain without correlation to the standing position. The diagnosis of a right ovarian vein syndrome has been made “ex adiuvantibus” because the endovascular treatment had reduced the periuterine venous congestion and, together with them, the pielic and ureteral dilation [5]. On the right side, in particular, the ovarian vein had an evident dilation in absence of blood reflux, tightly compressing the lumbar ureter that was deformed and irregular. The first description of the right ovarian vein syndrome has been made by Clark in the far 1965 [6]; by histological findings, he explained the true physiopathological mechanism subordinated to this pathology. A very close anatomic relationship between the right ovarian vein and the homolateral iliac artery generates a kind of vascular forceps that hold the lumbar ureter; its mucosal walls decubitus causes a fibrotic entrapment, and the chronic compression is testified by a plicar hypertrophia that is responsible of the aseptic leucocyturia. The phlogistic reaction may also cause hematuria. In our case, the hematuria, the first clinical sign, is thought to be a very rare demonstration of this syndrome and the vague right iliac pain, localized approximately to L4 level, is the result of the phlogistic involvement of the right lumbar ureter. The endovascular treatment [7–9] removed the congestion of the pelvic varicocele and, consequently, the detention of the right ovarian vein solved the ureteral compression [10, 11].
